# Evaluation of inflammation and follicle depletion during ovarian ageing in mice

**DOI:** 10.1038/s41598-020-79488-4

**Published:** 2021-01-11

**Authors:** Carolina Lliberos, Seng H. Liew, Pirooz Zareie, Nicole L. La Gruta, Ashley Mansell, Karla Hutt

**Affiliations:** 1 Department of Anatomy and Developmental Biology, Monash Biomedicine Discovery Institute, Monash University, Clayton, VIC Australia; 2 Department of Biochemistry and Molecular Biology, Monash Biomedicine Discovery Institute, Monash University, Clayton, VIC Australia; 3 Centre for Innate Immunity and Infectious Diseases, Hudson Institute of Medical Research, Clayton, VIC Australia

**Keywords:** Ageing, Inflammation, Reproductive biology

## Abstract

Reproductive ageing in females is defined by a progressive decline in follicle number and oocyte quality. This is a natural process that leads to the loss of fertility and ovarian function, cycle irregularity and eventually menopause or reproductive senescence. The factors that underlie the natural depletion of follicles throughout reproductive life are poorly characterised. It has been proposed that inflammatory processes and fibrosis might contribute to ovarian ageing. To further investigate this possibility, we evaluated key markers of inflammation and immune cell populations in the ovaries of 2, 6, 12 and 18-month-old C57BL/6 female mice. We report that the decrease in follicle numbers over the reproductive lifespan was associated with an increase in the intra-ovarian percentage of CD4 + T cells, B cells and macrophages. Serum concentration and intra-ovarian mRNA levels of several pro-inflammatory cytokines, including IL-1α/β, TNF-α, IL-6, and inflammasome genes ASC and NLRP3, were significantly increased with age. Fibrosis levels, as determined by picrosirius red staining for collagen I and III, were unchanged up to 18 months of age. Collectively, these data suggest that inflammation could be one of the mechanisms responsible for the age-related regulation of follicle number, but the role of fibrosis is unclear. Further studies are now required to determine if there is a causative relationship between inflammation and follicle depletion as females age.

## Introduction

As women age, the ability to conceive decreases, and the risk of miscarriage and birth defects increase. This decline in fertility is due to a reduction in the number and quality of oocytes stored in the ovaries, and is often referred to as ovarian ageing^1,2^. Females are born with a non-renewable ovarian reserve of approximately 1–2 million oocytes, which are housed in structures called primordial follicles^3,4^. Thereafter, the continuous activation of primordial follicles to begin folliculogenesis leads to the gradual depletion of the pool, resulting in loss of fertility and ovarian function, cycle irregularity and eventually menopause. Primordial follicles can also be directly lost from the ovarian reserve as a consequence of oocyte death and follicle attrition^2,3^. The specific factors that contribute to the reduced availability of healthy oocytes as women age are poorly characterised. However, with more women delaying childbearing until their later reproductive years^5^, there is a growing need to elucidate the mechanisms underlying the ovarian ageing process.

Inflammaging describes the chronic, sterile, low-grade inflammation that develops with age^6^. This process accelerates biological ageing and is associated with a host of age-related pathologies, including arthritis, type-II diabetes, chronic kidney disease, artherosclerosis, osteoporosis, Alzheimer’s disease, dementia and certain forms of cancer^7,8^ Inflammaging is often characterised by activation of the NLRP3 inflammasome in innate immune cells such as macrophages, and relatively subtle increases in pro-inflammatory cytokines, such as IL-6, TNF-α, IL-1β and IL-18^8–12^. Nucleotide-binding domain and leucine rich repeat containing family, pyrin domain containing 3 (NLRP3) inflammasome is a sensor of Danger Associated Molecular Patterns (DAMPS), recognising products released from damaged cells such as ATP and uric acid to initiate inflammation^8^. Upon recognising DAMPS, NLRP3 engages the adaptor molecule apoptosis-associated speck-like protein containing a CARD (ASC), forming oligomers that recruit and auto-catalytically mature the protease Caspase 1. This activated inflammasome complex enzymatically processes the pro-IL-1β and pro-IL-18 precursors into their respective mature pro-inflammatory cytokines IL-1β and IL-18, leading to their subsequent secretion from cells^8^. In mice, disrupting the NLRP3 inflammasome reduces inflammation and cytokine expression, extends the healthy lifespan and slows down the ageing process^13^. Importantly, the danger signals that activate the NLRP3 inflammasome are known to accumulate during ageing, thus causing a persistent inflammatory condition in aged people^6,8^.

Interestingly, recent studies in mice have revealed that the ovarian tissue microenvironment undergoes considerable changes during reproductive aging, which collectively, are suggestive of a pro-inflammatory phenotype. Using Picrosirius Red to label collagen I and III fibres, and an assay for hydroxyproline, Briley et al. reported an increase in stromal fibrosis in ovaries from animals of advanced reproductive age compared to reproductively young adults^14^. Furthermore, the ovaries of reproductively aged mice exhibited elevated levels of IL-1β and TNF-α mRNA and multi-nucleated macrophage giant cells were detected in the ovarian stroma^14^. Consistent with these observations, Zhang et al. found that many immune-related genes were upregulated in the ovaries of older mice, and detected an age-related change in the phenotype of intra-ovarian macrophage populations^15^. These data point to a relationship between inflammation and ovarian ageing. However, whether inflammation contributes to, or is a consequence of, the ovarian ageing process, is yet to be determined.

Inflammatory processes play a key role in many physiological reproductive events, including menstruation, embryo implantation and the onset of labour^16^. Within the ovary, macrophages, and inflammatory cytokines (which may be produced by immune cells or ovarian cells), such as TNF-α, IL-1, IL-6 and IL-8, modulate the secretion of steroid hormones necessary for follicle growth, and are essential for ovulation and the development and regression of the corpus luteum^16–20^. Proper ovarian function requires that these inflammatory processes are tightly regulated and appropriately resolved. Mounting evidence shows that unregulated and chronic inflammation contributes to pathologies of female reproductive function^21–23^. Recent studies suggest that chronic inflammatory conditions, such as Crohn’s disease and polymyositis, are associated with diminished ovarian reserves in women^24–26^. In addition, it has been suggested that obesity-dependent inflammation in the ovary could have an impact on ovarian function and oocyte quality^27^. These works indicate that there is a need to better understand the relationship and identify the inflammatory mechanisms between chronic inflammation, ovarian reserve and oocyte quality.

In this study, we expanded on the work of Briley and Zhang by first accurately determining follicle numbers and assessing the systemic and local ovarian inflammatory phenotype, followed by the identification and quantitation of immune cell populations in ovaries across the reproductive lifespan, all in a single cohort of C57BL/6 mice. Determining if increased inflammatory processes coincide with follicle depletion is the first step towards establishing a causal relationship. A comprehensive understanding of the mechanisms underlying reproductive ageing will, in the long term, assist in the development of new strategies to improve reproductive health for women as they age.

## Materials and methods

### Animals

Female C57BL/6 mice were used in this study because this strain is widely used for studies of ovarian biology and is the most common background of genetic mouse models (i.e. gene knockouts/mutants). Mice were housed in a temperature-controlled high-barrier facility (Monash University ARL) with free access to mouse chow and water, under a 12 h light–dark cycle. All animal procedures and experiments were performed in accordance with the NHMRC Australian Code of Practice for the Care and Use of Animals and approved by the Monash Animal Research Platform Animal Ethics Committee. Animals were bred to the age of 2 (young sexually mature) (n = 19), 6 (peak fertility) (n = 17), 12 (reproductively old) (n = 18) and 18 months (extreme reproductively aged) (n = 18) and then were humanely killed by isoflurane inhalation, followed by peripheral blood collection from the inferior vena cava. Serum was obtained, post coagulation and separated by centrifugation at 5000 rpm for 5 min, and then stored at − 80 °C. One ovary from each mouse was either fixed in Bouin’s solution, formalin, frozen at − 80 °C or directly used for Flow Cytometry. Stage of estrous cycle of animals used for Flow Cytometry was monitored by vaginal cytology immediately after sacrifice and recorded in Supplemental Table 1. Slides containing vaginal smears were stained with Rapid Diff Stain Kit (Australian Biostain, ARD.K) and classified based on the presence of different cell types, as previously described^28^. For Bouin’s and formalin procedures, samples were fixed overnight at 4 °C, and washed with 70% ethanol the following days. Body weights were measured prior to the sacrifice. No animals were used in this study if they had evidence of skin lesions or significant weight loss.

### Gene expression analysis

For gene expression analysis, one frozen ovary from each mouse was placed in RLT buffer (RNeasy Mini kit, Qiagen) containing β-mercaptoethanol (Sigma-Aldrich, M6250) and the tissue was homogenized using a Retsch mixer Mill MM 400. Total RNA was extracted using RNeasy Mini kit (Qiagen, 74104) followed by a DNase I treatment (Qiagen, 79254) to ensure the removal of any DNA contamination. Quantification of RNA concentration and purity was measured using the NanoDrop 2000 spectrophotometer (ThermoFisher Scientific). Finally, a total of 500 ng of RNA were reverse transcribed to cDNA using a SuperScript III First-Strand synthesis kit (ThermoFisher Scientific, 18080051). A Bio-Rad CFX384 machine was used for quantitative real-time PCR using the 2 × QuantiNova SYBR Green PCR Master Mix as instructed by the manufacturer (Qiagen, 208052). GAPDH was used as the endogenous gene and gene expression levels were calculated using the 2-ΔΔCt method. Results are expressed as fold change in expression compared to young mice (2-month-old mice). The thermal cycling conditions for 2 × QuantiNova SYBR Green PCR Master Mix were 2 min at 95 °C, followed by 40 cycles of 5 s at 95 °C and 10 s at 60 °C. The specificity of the process was controlled by Melting Curve analyses. The list of primers used in this study is shown in Supplemental Table 2.

### Western blot

One frozen ovary per mouse was placed in RIPA buffer (10 mM Tris–HCl pH 7.5, 1 mM EDTA, 0.5 mM EGTA, 1% Triton X-100, 0.1% SDS, 0.1% Sodium Deoxycholate, 140 mM NaCl supplemented with 1 mM of PMSF (Sigma-Aldrich, P7626) and 1 × Protease Inhibitor Cocktail (Sigma-Aldrich, 4693124001)) and the tissue was homogenized using a Retsch mixer Mill MM 400. Cell debris was removed by centrifugation (14,000 rpm for 15 min). The DC Protein Assay (Bio-Rad, 5000112) was used to determine the protein concentrations using bovine serum albumin as standard (Sigma-Aldrich, A9418). Briefly, 50 µg of each protein sample was loaded onto NuPAGE 4–12% Bis–Tris gel (ThermoFisher Scientific, NP0335BOX) and run at 200 V for 60 min. Protein was transferred to a polyvinylidene fluoride (PVDF) membrane (Sigma-Aldrich, GERPN1416LFP) using a wet electroblotter (XCell II Blot Module, ThermoFisher Scientific, EI9051). The membranes were probed with antibodies against NLRP3 (1:500; Novus Biologicals, NPB2-12446), ASC (1:500; Novus Biologicals, NBP1-78978), IL-6 (1:200; Santa Cruz Biotechnology, sc-57315), IL-1β (1:1000; Novus Biologicals, NB600-633), TNF-α (1:500; Novus Biologicals, NBP1-19532), IL-18 (1:500; ThermoFisher Scientific, PA5-81413), cleaved caspase-1 (1:1000 dilution, Cell Signaling, 89332), collagen I (1:1000, Abcam, ab34710) and β-actin (1:500; Sigma-Aldrich, A2066). Signals were detected by secondary HRP-conjugated antibodies: anti-rabbit (1:5000, ThermoFisher Scientific, 656120) and anti-mouse (1:2500, Promega, W4028) and enhanced chemiluminescence using ECL Prime Western Blotting Detection Reagent (Sigma-Aldrich, RPN2236). β-actin and total protein loading were both used to normalize the results. Full length Western blots are shown in Supplemental Fig. 4. Quantification was done using a Bio-Rad ChemiDoc MP Imaging System.

### Histology

Ovaries were fixed with 10% neutral-buffered formalin for 24 h, processed in 70% ethanol, embedded in paraffin and serially sectioned at 5 µm onto superfrost slides for Picrosirius staining. Bouin’s-fixed ovaries were embedded and processed in Glycol Methacrylate (GMA) embedding medium and serially sectioned at 20 µm. Every third section was collected onto plain glass slides, stained with Periodic Acid-Schiff (PAS) and counterstained with hematoxylin for follicle quantification.

### Assessment of primordial and primary follicles

Unbiased stereology was used to estimate the total number of primordial and primary follicles according to Myers et al.^29^. Stereology is considered the best-practise method for the unbiased and accurate quantification of cells in tissue sections as previously described^30–32^. In short, stereological quantification was performed using an Olympus BX50 microscope (Tokyo, Japan) mounted with an Autoscan stage (Autoscan Systems Pty Ltd, Melbourne, Australia), employing a × 100 oil immersion objective, which was controlled by the StereoInvestigator stereological system (Version 2018.2.2, MBF Bioscience 2015, MicroBrightField, Inc., Williston, Vermont). Every third section was counted, and estimation of total primordial and primary follicle numbers were determined by multiplying the raw counts (Q) by all three sampling fractions (1/f1, 1/f2 and 1/f3)^29^. Importantly, follicles were classified according to Myers et al.^29^ and were only counted when the oocyte nucleus was clearly visible.

### Quantification of secondary, antral and atretic follicles, and corpora lutea

Every ninth 20-µm PAS-stained resin section was used to estimate the total number of growing and atretic follicles under a Nikon light microscope using a 10 × objective. Follicles were identified according to Myers et al.^29^. Follicles were counted if the oocyte nucleus was clearly visible. Secondary follicles were classified by the presence of more than one layer or granulosa cells and the absence of an antral cavity; antral follicles were identified by the presence of small antrum or a large antral space; and atretic follicles were defined by more than 10% of granulosa cells appeared pyknotic (indicative of apoptosis). The number of corpora lutea was determined across sections encompassing the whole ovary to avoid double counting. Total secondary and antral follicle numbers were obtained by multiplying the sum of the raw follicle count for each section of a single ovary by nine as every ninth section were counted. Raw counts of corpora lutea were not adjusted.

### PicroSirius red staining

Paraffin embedded sections were deparaffinised in histolene, rehydrated in an ethanol gradient (100, 70 and 30%) and then immersed in a PicroSirius staining solution (0.1% w/v of Sirius Red F3BA in a saturated aqueous solution of picric acid) for 1 h at room temperature. After the incubation, the slides were washed three times in a solution of 0.5% glacial acetic acid. Sections were rapidly dehydrated using 100% ethanol, immersed in histolene and finally mounted using DPX mounting medium (Sigma-Aldrich, 100579). Mounted slides were dried overnight, and whole tissue section images were captured on the DotSlide system at × 10 magnification using an Olympus XC10 camera located in the Monash Micro Imaging facility (MMI) (Clayton, Australia). The area of positive staining was quantified as previously described^14^. Briefly, ImageJ was used to quantify the area of positive stained ovarian tissue above a threshold that was set based on the staining in the oldest animal. The same threshold value was used for all images analysed.

### Cytokine analysis

Serum IL-6, TNF-α and IL-18 levels were measured by a specific murine ELISA kit (Biolegend, 431307 (IL-6) and 430907 (TNF-α) and Abcam, ab216165 (IL-18)) according to manufacturer’s instructions. Serum samples were assayed in duplicates and the absorbance was measured at 450 and 570 nm using the CLARIOstar microplate reader (BMG Labtechset, Ortenberg, Germany).

### Flow cytometric analysis

Fresh ovaries were dissected and processed for cell isolation. Briefly, mouse tissues were placed in ice-cold Hank’s Balanced Salt Solution (HBSS). Ovaries were dissected and minced carefully with scissors. The disrupted tissue was digested in a 37 °C shaker at 120 rpm in digestion buffer (Dulbecco’s phosphate-buffered saline (DPBS, ThermoFisher Scientific, 14190250) with 0.4% Collagenase IV (Sigma-Aldrich, AC5138), 0.1% Deoxyribonuclease I (Sigma-Aldrich, DN25), 0.2% Dispase II (Sigma-Aldrich, D4693) and 0.2% Hyaluronidase (Sigma-Aldrich, H3506) for 30 min. Digestion was stopped by adding 1 ml of neutralisation buffer (DPBS containing 20% dialysed FBS (Assay Matrix, ASFBS-U) and 5 mM EDTA). Cell pellet was collected by centrifugation (3300 rpm for 4 min at 4 °C), resuspended in FACS buffer (PBS containing 0.5% Bovine Serum Albumin (BSA, Sigma-Aldrich, A9418) and 5 mM EDTA), filtered through a 35 μm nylon mesh filter (Corning, 352235) and counted.

For staining, 3 × 10^5^ ovarian cells/ml were blocked with an Fc block (anti-CD16/CD32 mAb (clone 2.4G2)) for 10 min on ice. Cells were then incubated with the appropriate antibodies for 30 min on ice and stained with Live/Dead Viability Stain BD Horizon FVS-700 (FVS700, diluted in PBS) for 10 min on ice. Cells were finally transferred to round bottom polypropylene FACS tubes (Falcon). FACS buffer was used as the diluent for each stain unless indicated otherwise. For the detection of immune cells, antibodies against CD19, CD4, CD8a, CD11b, F4/80, NK1.1 and TCRβ were used. Details of the antibodies and other stains are specified in Supplemental Table 3. Data was collected on a BD LSRFortessa X-20 cell analyser (BD Biosciences, New Jersey, USA) and analysed using FlowJo version 10 (Tree Star Inc., Oregon, USA). Live cells were gated using FSC and SSC before analysis of all immune cell subsets.

### Statistical analysis

Data are presented as mean ± standard error of the mean (SEM) and statistical analyses were undertaken using GraphPad Prism version 8 (GraphPad Software, California, USA). Normally distributed data were analysed using unpaired Student’s t-test to compare two groups and one-way analysis of variance (ANOVA) with Tukey’s post hoc test was used for multiple comparisons. Data normality was evaluated using Shapiro–Wilk test. Data that did not follow a normal distribution were analysed by Mann–Whitney test for pairwise comparisons, and Kruskal–Wallis test to compare multiple groups. Differences were considered statistically significant when *p* < 0.05.

## Results

### Follicle numbers decline with age in the mouse ovary

**Figure 1:**
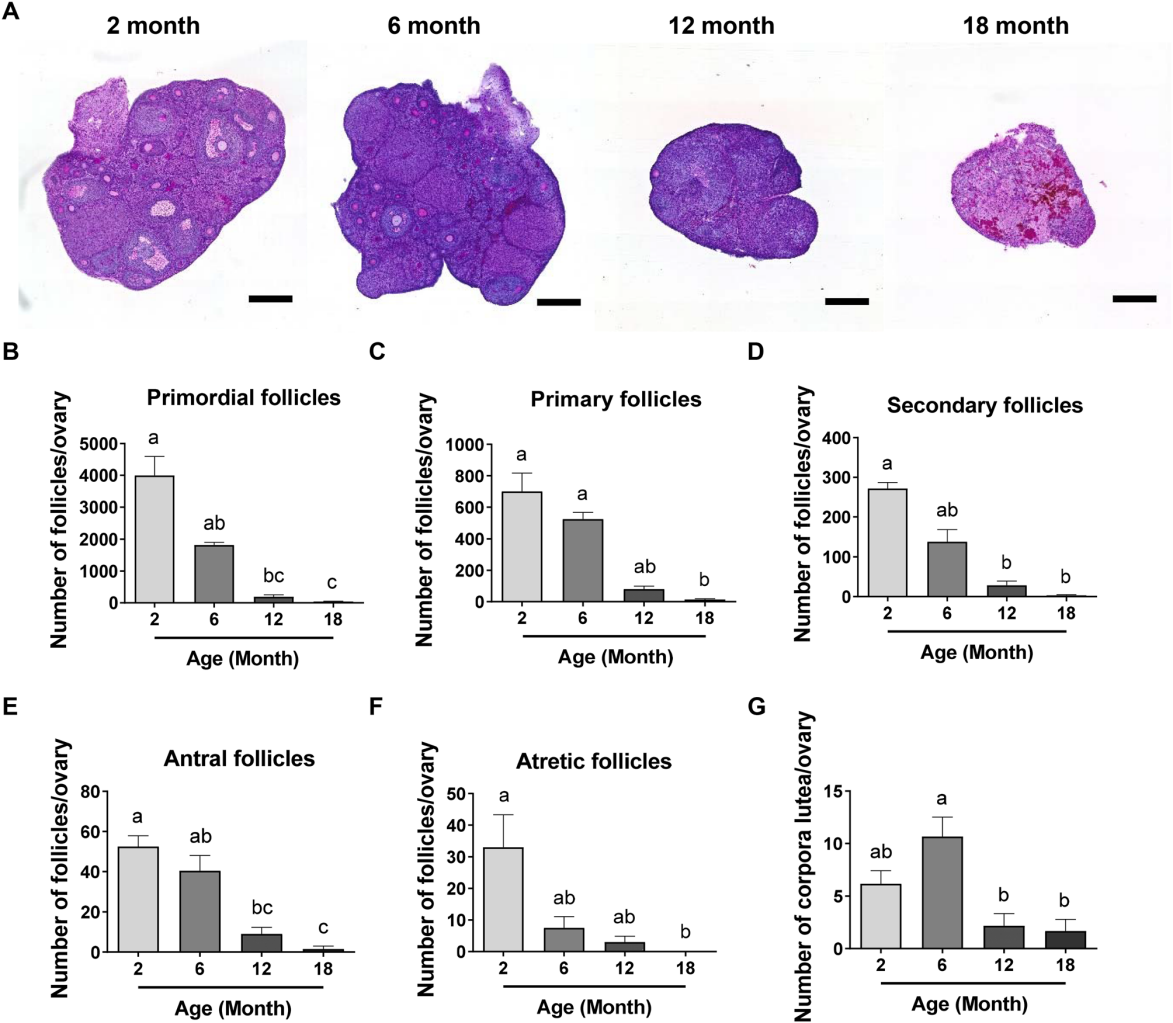
Reproductive ageing is associated with a progressive loss of follicle and corpora lutea numbers in the mouse ovary. (**A**) Representative images of PAS-stained ovarian sections from 2, 6, 12 and 18-month-old mice visualized by bright-field microscopy. Scale bars are 400 μm. Primordial (**B**), primary (**C**), secondary (**D**), antral (**E**), atretic follicle (**F**) and corpora lutea (**G**) numbers in ovaries from 2, 6, 12 and 18-month-old mice. n = 6 per cohort. Data are presented as mean ± SEM; Kruskal–Wallis test, Dunn’s multiple comparisons test: a, b and c are significantly different among groups (*p* < 0.05); Ordinary One-way ANOVA, Tukey’s multiple comparisons test: a, b and c are significantly different among groups (*p* < 0.05).

To characterise the progression of follicle depletion throughout reproductive life, follicle numbers were quantified in ovaries from 2, 6, 12 and 18-month-old C57BL/6 female mice (Fig. 1A–F; Supplemental Fig. 1A–E,G). At two months of age, C57BL/6 mice are sexually mature young adults. At this age, ovaries contained abundant numbers of primordial follicles (3998 ± 599) (Fig. 1B). As expected, the number of primordial follicles were lower in older animals (Fig. 1B). At 6 months of age, the numbers of primordial follicles were 50% of that observed at 2 months, 1817 ± 87.3 (Fig. 1B). In reproductively aged 12-month-old mice, very few primordial follicles remained (190.7 ± 61.93), and at 18 months of age, when mice are infertile, ovaries were almost completely devoid of primordial follicles (40 ± 11.37) (Fig. 1B). A similar age-related decline in numbers was observed for primary, secondary and antral follicles, as well as for atretic follicles (Fig. 1C–F). Primary follicle numbers were similar at 2 and 6 months of age (700.3 ± 116.4 and 524.8 ± 42.57, respectively), and decreased significantly at 12 and 18 months (80 ± 19.35 and 13.5 ± 6.037, respectively) (Fig. 1C). Secondary follicle counts at 6 months (138 ± 30.71) exhibited a half-fold change compared to that observed at 2 months (271.5 ± 15.31) (Fig. 1D). Regarding the number of antral follicles, we counted 52.5 ± 5.408 and 40.5 ± 7.619 follicles per ovary at 2 and 6 months of age, respectively, decreasing significantly in 12 and 18-month-old mice (9 ± 3.286 and 1.5 ± 1.5, respectively) (Fig. 1E). Finally, atretic follicle numbers exhibited a trend towards a decrease at 6 and 12 months (7.5 ± 3.612 and 3 ± 1.897, respectively) compared to 2 months (33 ± 10.31 follicles), and this reduction was significant in 18-month-old ovaries (Fig. 1F).

Corpora lutea numbers were also determined (Fig. 1G; Supplemental Fig. 1F), as an indication of ovulatory activity. The number of corpora lutea was similar at 2 and 6 months of age (6.167 ± 1.249 and 10.67 ± 1.856, respectively), when mice are reproductively active and ovulating. In ovaries from 12 and 18-month-old mice, corpora lutea were significantly fewer (2.167 ± 1.167 and 1.667 ± 1.116, respectively) than in 6-month-old mice, consistent with loss of reproductive capacity and infertility (*p* = 0.0324 and *p* = 0.0193, respectively) (Fig. 1G).

### Fibrosis does not change in the ageing mouse ovary

**Figure 2:**
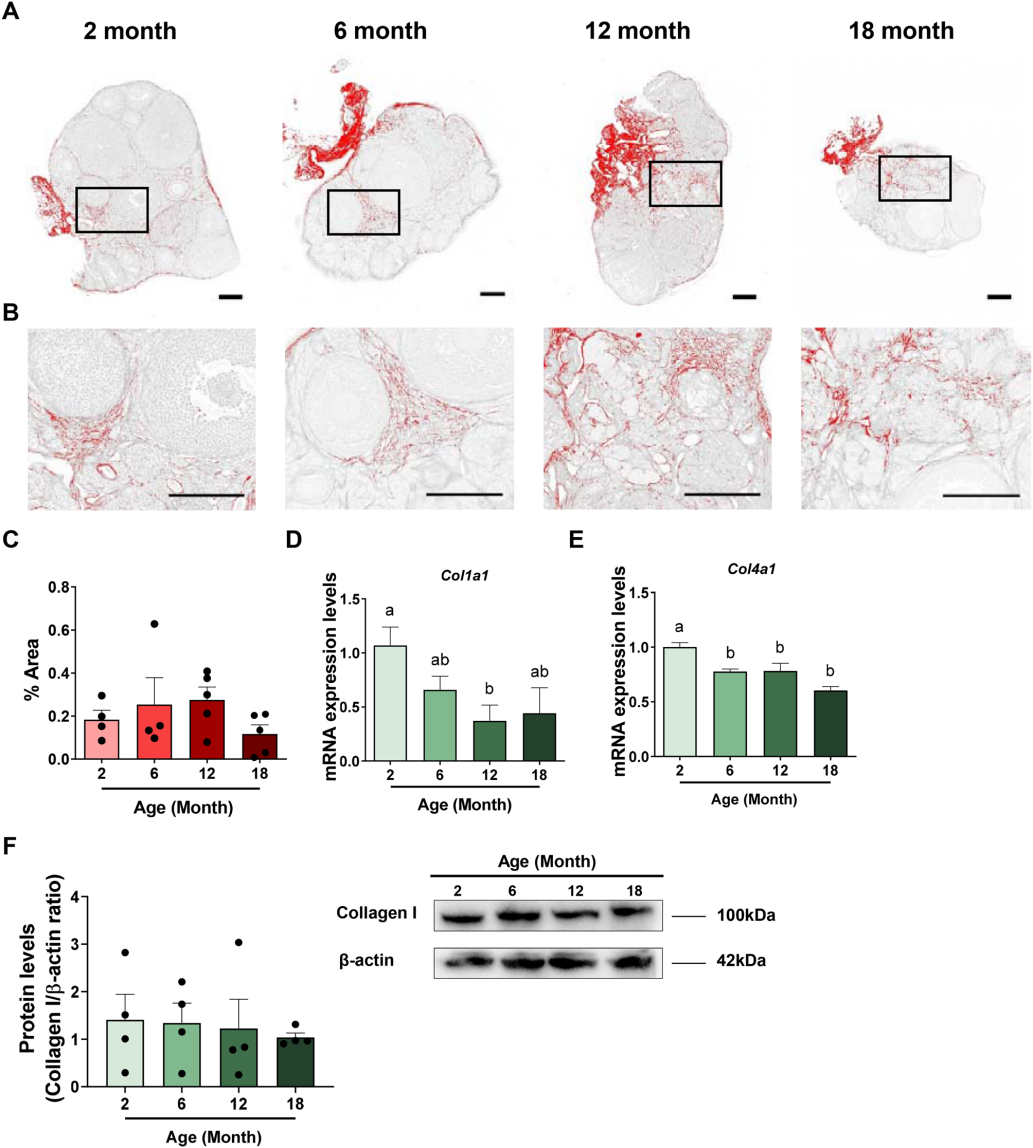
Evaluation of ovarian fibrosis during reproductive ageing. (**A**) Representative processed color threshold images of PicroSirius Red (PSR)-stained ovarian tissue sections used to quantify fibrosis in 2, 6, 12 and 18-month-old mice. (**B**) Higher magnification images from specific regions boxed in ovaries from 2, 6, 12 and 18-month-old mice. Scale bars are 200 μm. (**C**) Percentage of PSR-positive staining in ovaries from 2, 6, 12 and 18-month-old mice. n = 4–5 per cohort. Gene expression levels of Col1a1 (**D**) and Col4a1 (**E**) in ovaries from 2, 6, 12 and 18-month-old mice. n = 4–6 per cohort. (**F**) Quantification and representative image of ovarian protein levels of collagen I from 2, 6, 12 and 18-month-old mice. n = 4 per cohort. Data are presented as mean ± SEM; C: Kruskal–Wallis test, Dunn’s multiple comparisons test (*p* > 0.05). (**D**,**E**,**F**): Ordinary One-way ANOVA, Tukey’s multiple comparisons test: a and b are significantly different among groups (*p* < 0.05). Each dot represents one animal.

Increased collagen deposition and fibrosis can be the end result of chronic inflammatory reactions. Therefore, to determine if fibrosis is associated with ovarian ageing, we assessed ovarian fibrosis during reproductive ageing in 2, 6, 12 and 18-month-old C57BL/6 mice using Picrosirius Red (PSR) staining (Fig. 2A-C). PSR is a histological stain that binds specifically to collagen I and III^14^. It is commonly used to evaluate the level of fibrosis in tissue sections and increased overall levels have been reported in very aged ovaries^14^. As expected, PSR staining was detected in thecal layers surrounding follicles, in the ovarian stroma, and was also associated with surface epithelium at 2, 6 and 12 months of age (Fig. 2A,B). Staining in the stroma and epithelium at 18 months was similar (Fig. 2A,B). The percentage of PSR-positive stained area did not significantly change during ovarian ageing (Fig. 2C).

To evaluate how the ovarian microenvironment changes during ageing, we further investigated mRNA and protein levels of collagen I (Col1a1) and the mRNA levels of collagen IV (Col4a1) in ovaries from 2, 6, 12 and 18-month-old mice (Fig. 2D–F). Type I and IV collagen are two of the most commonly examined ECM factors and both contribute to fibrosis^33^. The expression of both genes exhibited an age-related decrease with ageing ovary. While Col1a1 mRNA expression levels were significantly lower in 12-month-old mice compared to ovaries from younger mice (Fig. 2D), protein levels were unchanged across the age groups (Fig. 2F). Col4a1 mRNA was significantly decreased at 6, 12 and 18 months relative to 2-month-old mice (Fig. 2E).

### Reproductive ageing is associated with a pro-inflammatory microenvironment in the mouse ovary

**Figure 3:**
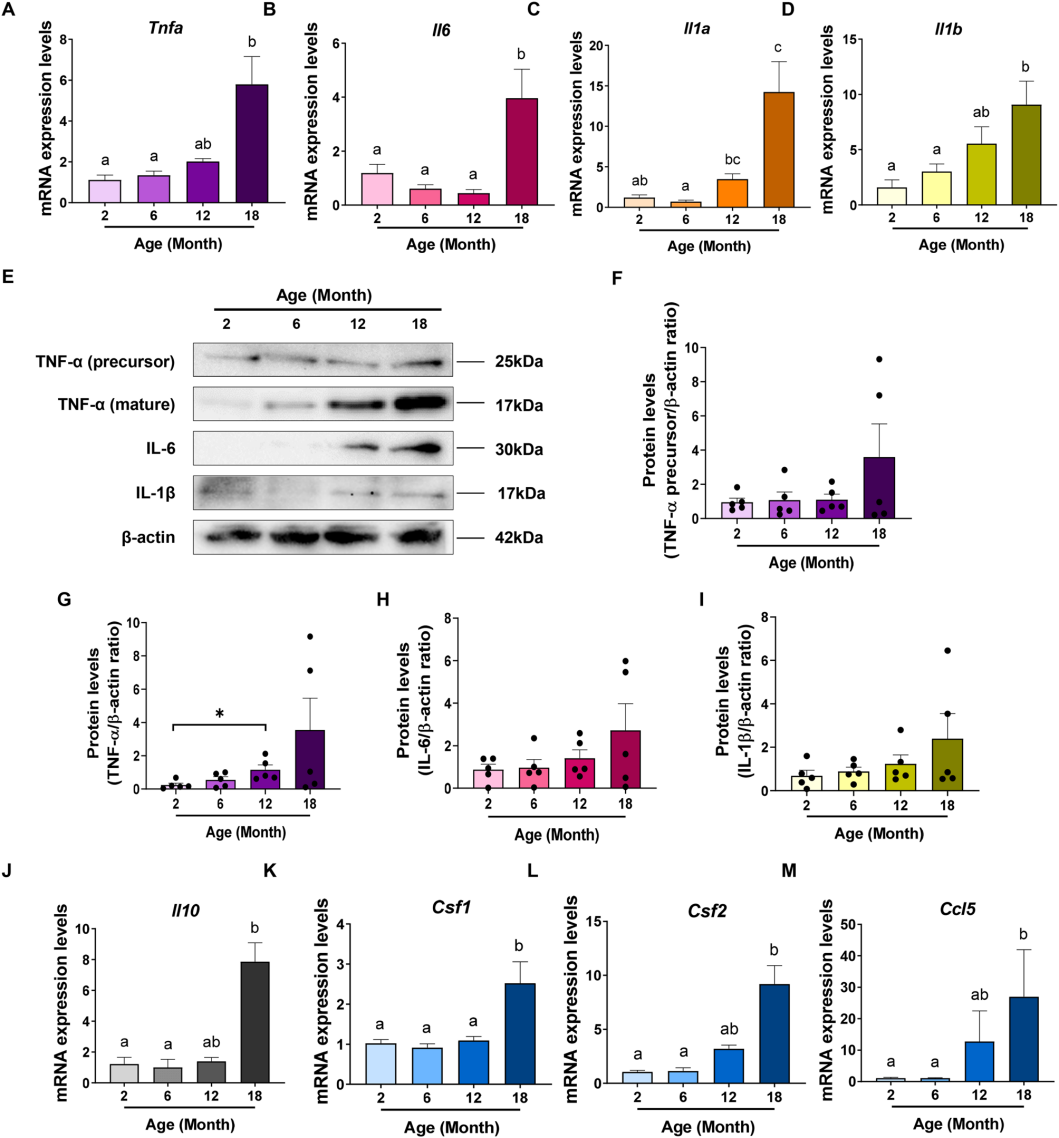
Reproductive ageing is associated with a local inflammatory microenvironment in the mouse ovary. Gene expression levels of Tnfa (**A**), Il6 (**B**), Il1a (**C**) and Il1b (**D**) in ovaries from 2, 6, 12 and 18-month-old mice. n = 6 per cohort. Data are presented as mean ± SEM; (**A**) and (**C**): Kruskal–Wallis test, Dunn’s multiple comparisons test: a, b and c are significantly different among groups (*p* < 0.05); (**B**) and (**D**): Ordinary One-way ANOVA, Tukey’s multiple comparisons test: a and b are significantly different among groups (*p* < 0.05). (**E**) Representative image of a Western blot showing ovarian protein levels of TNF-α (precursor), TNF-α (mature), IL-6 and IL-1β from 2, 6, 12 and 18-month-old mice. Quantification of protein levels of TNF-α (precursor) (**F**), TNF-α (mature) (**G**), IL-6 (**H**) and IL-1β (I) in ovaries from 2, 6, 12 and 18-month-old mice. n = 5 per cohort. Data are presented as mean ± SEM; Mann–Whitney test (*p < 0.05). Each dot represents one animal. mRNA levels of Csf1 (**J**), Csf2 (**K**), Ccl5 (**L**) and Il10 (**M**) in ovaries from 2, 6, 12 and 18-month-old mice. n = 6 per cohort. Data are presented as mean ± SEM; I: Ordinary One-way ANOVA, Tukey’s multiple comparisons test: a and b are significantly different among groups (*p* < 0.05); (**J**),**K**,**L**): Kruskal–Wallis test, Dunn’s multiple comparisons test: a, and b are significantly different among groups (*p* < 0.05).

A progressive trend towards a pro-inflammatory cytokine profile is a marker of mammalian ageing^34^. To investigate the possibility that ovarian ageing is similarly associated with an increase in pro-inflammatory status, we examined the mRNA expression levels of major pro-inflammatory genes Tnfa, Il6, Il1a and Il1b in ovaries from 2, 6, 12 and 18-month-old mice (Fig. 3A–D). Overall, there was a trend towards increasing expression level with age for each of these genes. Notably, all genes exhibited significantly higher expression levels in ovaries from 18-month-old mice relative to 2 and 6-month-old mice.

To further examine the pro-inflammatory profile of the ageing ovary, protein levels of TNF-α precursor and mature TNF-α, IL-6 and IL-1β were assessed in ovaries from 2, 6, 12 and 18-month-old mice by Western blot (Fig. 3E–I). Similar to the results for mRNA, a trend towards increased protein levels of these pro-inflammatory cytokines was observed with age, but differences were not statistically significant when analysed by one way ANOVA followed by Tukey’s post hoc test for multiple comparisons (*p* > 0.05), likely due to the large amount of inter-animal variability. However, an analysis using t-test revealed that levels of mature TNF-α protein were significantly higher in ovaries from 12-month-old mice when compared to 2-month-old mice (*p* = 0.0159) (Fig. 3G). mRNA levels of IL-10, an anti-inflammatory cytokine, were also measured (Fig. 3J) and a significant increase in the expression levels of Il10 in 18-month-old relative to 2 and 6-month-old mice was detected. IL-10, like TNF-α, IL-1 and IL-6, is an NF-κB-dependent gene ^35^, thus increased expression of IL-10 could be indicative of the activation of NF-kB signalling.

We next examined the gene expression pattern of Csf1, a cytokine involved in the proliferation and maturation of macrophages, and Csf2, which regulates both macrophages and granulocytes (Fig. 3K,L). Both Csf1 and Csf2 expression were significantly increased in ovaries from 18-month-old mice compared to 2 and 6-month-old mice. In addition, where Csf1 expression levels were consistent at 2, 6 and 12 months of age, expression levels of Csf2 showed an age-related trend towards an increase across these ages (Fig. 3L). Finally, we also assessed the gene expression profile of Ccl5, a chemokine involved in leukocyte recruitment to sites of inflammation, and saw a similar trend towards an age-related increase at 2, 6 and 12 months, and expression levels that were significantly higher at 18 months compared to 2 and 6 months (Fig. 3M).

### NLRP3 inflammasome

**Figure 4:**
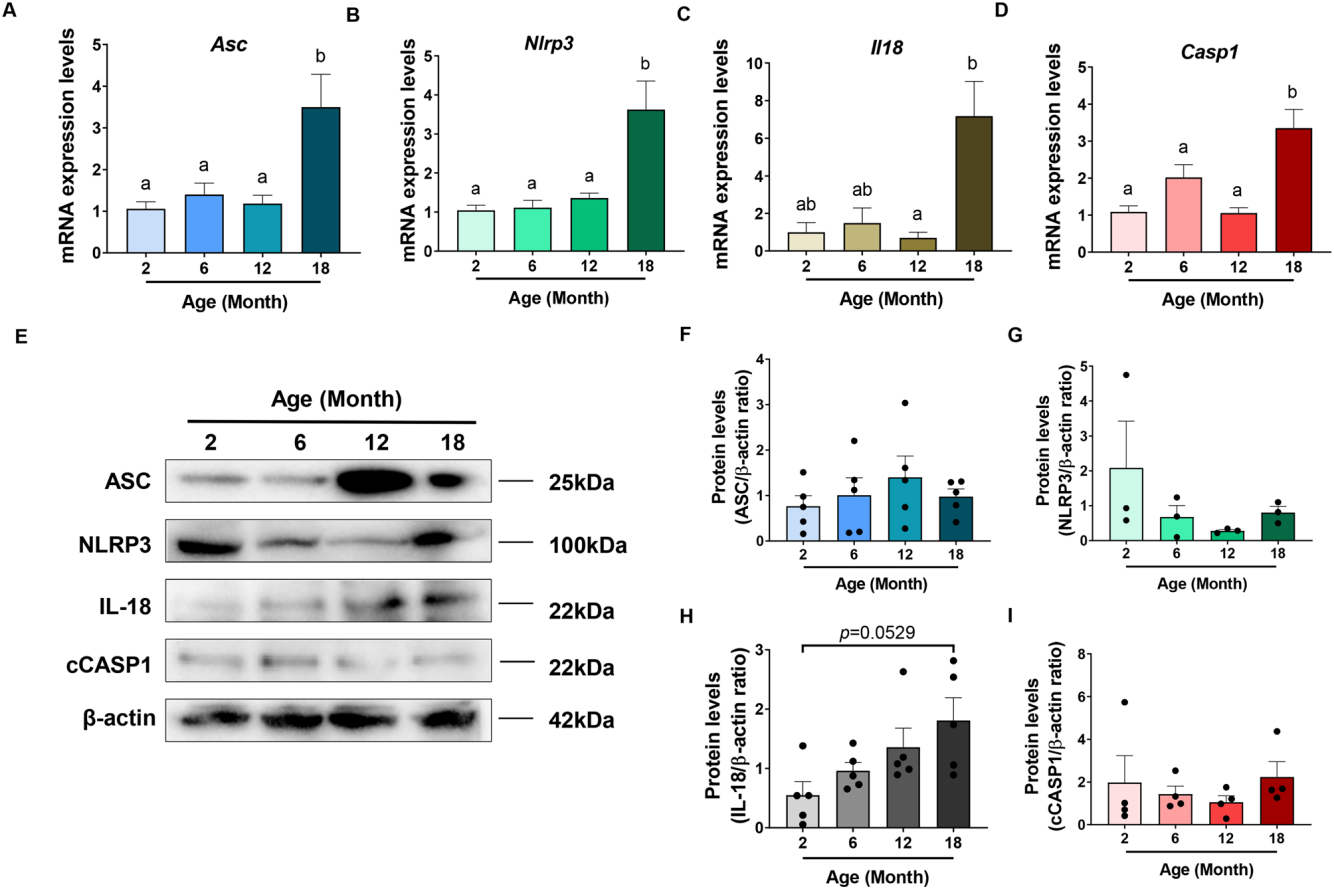
Reproductive ageing is accompanied by an increase in the intra-ovarian expression of inflammatory cytokines. Gene expression levels of inflammasome-related genes Asc (**A**), Nlrp3 (**B**), Il18 (**C**) and Caspase1 (Casp1) (**D**) in ovaries from 2, 6, 12 and 18-month-old mice. n = 6 per cohort. Data are presented as mean ± SEM; (**A**,**B**,**D**): Ordinary One-way ANOVA, Tukey’s multiple comparisons test: a and b are significantly different among groups (*p* < 0.05). (**C**): Kruskal–Wallis test, Dunn’s multiple comparisons test: a and b are significantly different among groups (*p* < 0.05). (**E**) Representative image of a Western blot showing ovarian protein levels of ASC, NLRP3, IL-18 and cleaved Casp1 (cCASP1) from 2, 6, 12 and 18-month-old mice. Quantification of protein levels of ASC (**F**), NLRP3 (**G**), IL-18 (**H**) and cCASP1 (**I**) in ovaries from 2, 6, 12 and 18-month-old mice. n = 3–5 per cohort. Data are presented as mean ± SEM; E, F, Ordinary One-way ANOVA, Tukey’s multiple comparisons test (*p* > 0.05). (**G**,**H**); Kruskal–Wallis test, Dunn’s multiple comparisons test (*p* > 0.05). Each dot represents one animal.

The inflammasome complex is a critical element of the innate immune system and NLRP3 inflammasome activation has been specifically associated with sterile inflammation during mammalian ageing^8,13^. To evaluate if inflammasome expression reflects changes with ovarian ageing, expression levels of the adaptor protein Asc and Nlrp3 were measured in ovaries from 2, 6, 12 and 18-month-old mice (Fig. 4A,B). Using qRT-PCR, mRNA levels were found to be stable at 2, 6 and 12 months, but an increase in Asc mRNA was observed in ovaries from 18-month-old mice when compared to 2, 6 and 12-month-old mice (Fig. 4A). Similarly, the expression levels of Nlrp3 were significantly elevated in 18-month-old mice compared to ovaries from younger mice (Fig. 4B). To further reveal a potential role for the NLRP3 inflammasome in ovarian ageing, the expression of Il18 and caspase-1 were analysed in ovaries from 2, 6, 12 and 18-month-old mice. IL-18 (together with IL-1β) is processed by the NLRP3 inflammasome via caspase 1 and mediates downstream pro-inflammatory effects^8^. Il18 expression levels were significantly increased in ovaries from 18-month-old mice compared to 12-month-old mice (Fig. 4C). Casp1 expression was significantly higher in 18-month-old mice relative to 2, 6 and 12-month-old mice (Fig. 4D).

Protein levels of inflammasome-associated proteins ASC and NLRP3 as well as inflammasome-related Caspase-1 (cleaved) and inflammatory cytokine IL-18, were also assessed in ovaries from 2, 6, 12 and 18-month-old mice by Western blot (Fig. 4E–I). Despite an increase in the Asc, Nlrp3 and Casp1 mRNA levels at 18 months of age, these proteins did not exhibit any statistically significant change during reproductive ageing (*p* > 0.05). However, inflammasome activity may be increased within the ageing ovary, as a trend towards an increase in the protein levels of IL-18 was observed in ovaries at 18 months of age (*p* = 0.0529) (Fig. 4H).

### Reproductive ageing is accompanied by an increase in the serum levels of inflammatory cytokines IL-6 and IL-18

**Figure 5:**
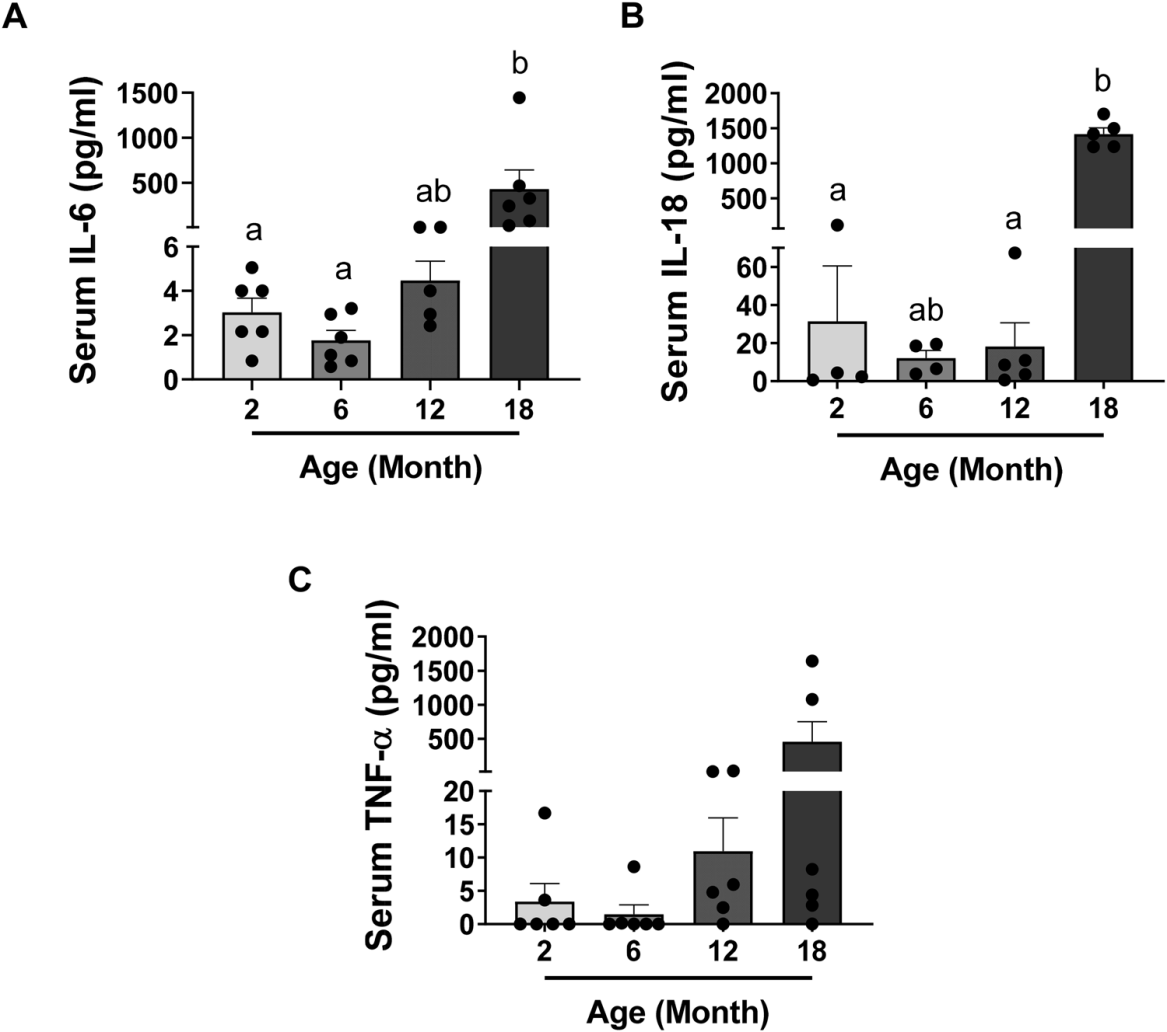
Reproductive ageing is accompanied by an increase in the serum levels of inflammatory cytokines IL-6 and IL-18. Serum IL-6 (**A**), IL-18 (**B**) and TNF-α (**C**) concentration measured in 2, 6, 12 and 18-month-old mice. n = 4–6 per cohort. Data are presented as mean ± SEM; Kruskal–Wallis test, Dunn’s multiple comparisons test: a and b are significantly different among groups (*p* < 0.05). Each dot represents one animal.

To assess the systemic inflammatory status of mice during reproductive ageing, the serum levels of pro-inflammatory cytokines IL-6, IL-18 and TNF-α were measured in mice at 2, 6, 12 and 18 months of age (Fig. 5). Levels of circulating IL-6 were dramatically increased in 18-month-old mice relative to 2 and 6-month-old mice (Fig. 5A). IL-18 serum concentration was also significantly higher in 18-month-old mice compared to 2 and 12 months of age (Fig. 5B). There was a trend towards an increase in serum TNF-α levels with age (Fig. 5C), but despite a very large increase in mean values between 2 and 18 months, this difference was not statistically significant due to large inter-animal variability (*p* > 0.05). Taken together, these results suggest increased systemic inflammation in aged females indicative of inflammaging, coinciding with infertility and reduced primordial follicles.

### Reproductive ageing is associated with an expansion of both innate and adaptive immune cell populations in the mouse ovary

**Figure 6:**
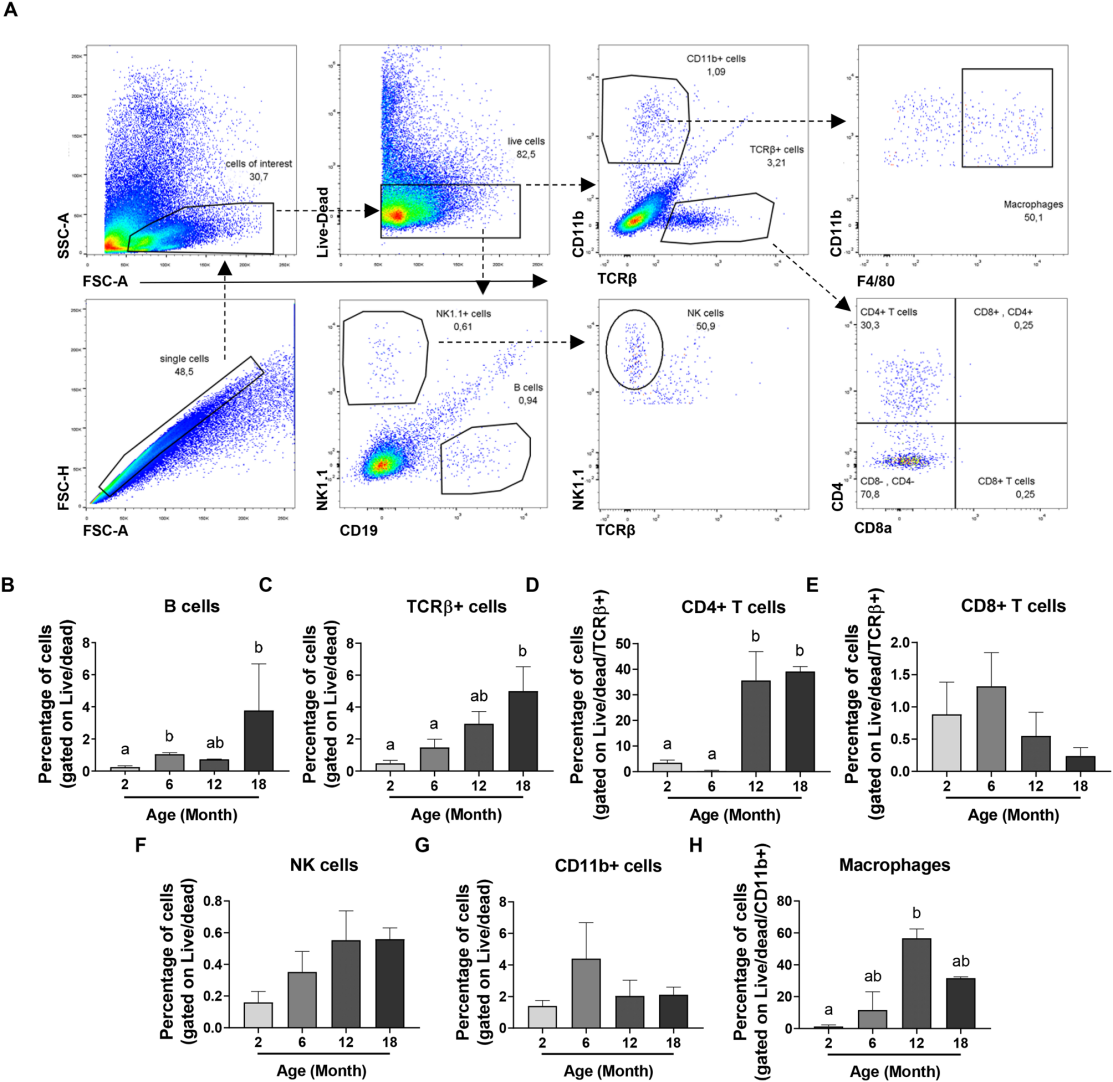
Ovarian immune cell populations increase during reproductive ageing. (**A**) Gating strategy used to identify different immune cell populations in ovaries from 2, 6, 12 and 18-month-old mice. Percentage of B cells (**B**), T cells (**C**), CD4 + (**D**) and CD8 + T cells (**E**), NK1.1 + TCRβ- NK cells (**F**), CD11b + cells (**G**) and F4/80 + CD11b + macrophages (**H**) present in ovaries from 2, 6, 12 and 18-month-old mice. n = 3–5 per cohort. Data are presented as mean ± SEM; (**B**,**F**,**G**,**H**): Kruskal–Wallis test, Dunn’s multiple comparisons test: a and b are significantly different among groups (*p* < 0.05); (**C**,**D**,**E**): Ordinary One-way ANOVA, Tukey’s multiple comparisons test: a and b are significantly different among groups (*p* < 0.05).

Given the tendency towards a systemic and intra-ovarian pro-inflammatory profile observed during reproductive ageing, and the critical role of IL-18 and IL-1β in maturing the adaptive response, we investigated if immune cell populations were altered in ovaries from 2, 6, 12 and 18-month-old mice using flow cytometry. The gating strategy is described in Fig. 6A. Briefly, ovarian cells were first gated for singlets (FSC-H vs FSC-A) and lymphoid/myeloid populations (SSC-A vs FSC-A). Then, taking only live cells, CD11b, TCRβ, NK1.1 and CD19 expression were used to identify different immune cell populations. NK cells were gated as NK1.1 + TCRβ- and B cells as CD19 + . T cells were identified on the basis of TCR expression (TCRβ +) and either CD4 or CD8. Finally, surface expression of F4/80 was used to identify macrophages within the CD11b + population (F4/80 + CD11b +).

There was a trend towards an increase in the proportion of NK cells in the aged ovary, but this was not statistically significant (Fig. 6F; Supplemental Fig. 2B). The percentage of B cells in the ovary was significantly increased in 6 and 18-month-old mice compared to 2-month-old mice (Fig. 6B; Supplemental Fig. 2A). Similarly, the percentage of T cells in the ovary was significantly increased in 18-month-old mice compared to 2 and 6-month-old mice (Fig. 6C; Supplemental Fig. 2C). Remarkably, the percentage of CD4 + T cells was significantly higher in ovaries from 12 and 18-month-old mice compared to 2 and 6-month-old mice (Fig. 6D; Supplemental Fig. 2E), which would explain the increase observed in the proportion of T cells (Fig. 6C). However, no significant change was observed in the percentage of CD8 + T cells with ovarian ageing (*p* > 0.05) (Fig. 6E; Supplemental Fig. 2E).

With regard to myeloid populations, the percentage of ovarian cells comprising the CD11b + population did not show any significant difference between the age groups analysed (*p* > 0.05) (Fig. 6G; Supplemental Fig. 2C). However, the percentage of F4/80 + CD11b + macrophages showed a significant increase in ovaries from 12-month-old mice compared to 2-month-old mice (Fig. 6H; Supplemental Fig. 2D). Altogether, these data suggest that the innate and adaptive immune systems are both altered within the ovary over the course of reproductive ageing. However, no significant differences were observed in the number of infiltrating cells in the ageing ovary (Supplemental Fig. 3).

## Discussion

In this study, we investigated the possibility that inflammation plays a critical role in physiological ovarian ageing. In mice, we demonstrate that the age-related decrease in primordial, primary, secondary and antral follicles, coincides with an increase in the intra-ovarian and systemic expression levels of major pro-inflammatory and inflammasome-related genes and proteins. In addition, proportion of immune cell populations were found to increase in ovaries from reproductively aged mice. These findings are consistent with a role for inflammation in ovarian ageing. However, further studies are required to establish a direct causative relationship between inflammation and the physiological depletion of follicles and loss of oocyte quality that occur as females age.

IL-1α, IL-1β, TNF-α, IL-6 and IL-18 are among the cytokines that are most commonly found to increase at the intra-organ and systemic levels during inflammaging^8,36^. Initially, we examined the ovarian mRNA expression levels of major pro-inflammatory cytokines Tnfa, Il6, Il1a and Il1b and observed an overall trend towards increasing expression level with increasing age. Notably, all genes exhibited significantly higher expression levels in ovaries from reproductively aged mice with depleted follicular reserves, relative to reproductively young mice with abundant follicles. Intra-ovarian protein levels for TNF-α precursor and mature forms, IL-6 and IL-1β showed a similar age-related trend. Overall, these results are consistent with previous studies reporting an association between ovarian ageing and an inflammatory ovarian microenvironment^14,15^. Notably, we also examined the systemic levels of IL-6, IL-18 and TNF-α and observed dramatic increases in these cytokines in serum 18-month-old mice, compared to 2-, 6-, and 12-month-old mice. These observations are consistent with prior literature reporting a strong association between theses cytokines and chronic inflammation during ageing^6,13,14,36,37^.

In particular, levels of serum IL-6 increase with age in humans, and may mediate fibrosis by upregulating the expression of collagen, or by promoting cytokines that support collagen production^9,38^. The ovary undergoes a dramatic and periodic remodelling of extracellular matrix (ECM) as a consequence of cyclic follicle development and ovulation and this is part of normal ovarian function^39^. However, excessive deposition of ECM components and ovarian fibrosis have been associated with decreased follicle density and ovarian dysfunction in women with ovarian endometriosis cysts suggesting that there may be a link between follicle depletion and fibrosis^39^. However, no changes in collagen I and III levels were observed in aged ovaries compared to young ovaries using PSR staining, suggesting an absence of fibrosis. Furthermore, an age-related decrease in collagen 1 and 4 mRNA levels was found by qRT-PCR. Type IV collagen is mostly present in the theca cell compartment, appearing in primary follicles and increasing with follicle development^33^. Therefore, this observed reduction is consistent with the follicle loss that occurs with age. Type I collagen is found throughout the stroma, in granulosa and theca cells of ovarian follicles and, in high levels, in the outer epithelial layer of the ovary^33^. The decreased observed in col1a1 expression levels at 12 and 18 months of age could also be associated with lower number of follicles present in the ovaries and also suggests that accumulation of collagen in the ovarian stroma might not occur with age. This observation is also supported by collagen I protein levels, which remain unchanged across reproductive lifespan. In contrast, Briley et al. showed a significant increase in PSR-positive stained area with ovarian ageing in CD1 and CB6F1 mice^14^. This discrepancy may be because the mice used in our study were of a different genetic background (C57BL/6) and the rates and propensity for collagen accumulation may vary between strains. It is also worth mentioning that the mice used for PSR analyses were not synchronised. While there are no literature suggesting that collagen I and III protein levels change across the estrous cycle in the mouse ovary, we cannot rule out the possibility that cycle differences in the younger mice could have reduced our ability to detect age related changes in PSR staining levels. Indeed, immunostaining of collagen I and IV showed that their expression in the mouse uterus change across estrous cycle^40^. Importantly, Briley et al.^14^ did not observe significant collagen accumulation in the ovaries until 18–22 months of age, whereas fibrosis at 12 months of age was similar to that observed at 4 months of age. These findings are consistent with our data.

While some of these data were not statistically significant, it is important to note that increases in cytokine levels in the context of inflammaging are often subtle (unlike the responses seen during acute infection, for example), but biologically meaningful none the less^8,41^. Small increases in cytokine expression, such as those we observed in ovaries from mice progressing from reproductively young (2 and 6 months) to reproductively older (12 months), are consistent with a transition to a low-grade pro-inflammatory environment, which could in part explain the rate of follicle loss observed during this time. Furthermore, long-term accumulation of persistent but small changes in cytokine expression could result in a significant change as observed at extreme end of reproductive aging at 18 months, where the inflammatory levels are high enough to reach statistical significance when compared to the rest of the cohort.

The NLRP3 inflammasome is considered to be a master driver of inflammaging^13,42^ as IL-1β and IL-18 themselves can drive inflammation via their capacity to activate the prototypic inflammatory transcription factor NF-κB^43,44^. Therefore, tonic concentrations of mature IL-1 β or IL-18 can induce chronic inflammation. Interestingly, we observed an increase in the mRNA expression for Asc and Nrlp3, together with downstream factors, caspase-1, IL-1β and IL-18 in ovaries reproductively aged mice, compared to younger mice. However, we were unable to detect an age-related increase in NLRP3 inflammasome protein levels, though a clear trend was observed for IL-18. Our inability to detected significant changes in protein levels even though changes were detected at the mRNA level could be due to immunoblots are less sensitive than qRT-PCR. Alternative, it may be that NLRP3 and ASC levels do not change in the ovary with age. In other ageing studies, however, renal NLRP3 protein levels did exhibit a significant increase in old rats compared to young^37^.

While the mechanisms by which age-associated inflammation might deplete follicle numbers are not yet clear, there is evidence in the literature that dysregulation of pro-inflammatory cytokines can cause follicle death. Several sources of TNF-α in the ovary have been described, including granulosa cells, theca cells or macrophages, along with many different functions of this cytokine, indicating the importance of TNF-α on ovarian function^17,18,45^. Kaipia et al. 1996 showed that TNF-α stimulates apoptotic DNA fragmentation and apoptosis in ovarian follicles in vitro^46^. Morrison et al. 2002 also demonstrated that TNF-α was able to promote oocyte death during the early stages of follicular development in rats^47^. Furthermore, Greenfeld et al. 2007 indicated that TNF-α was cytotoxic to immature oocytes in mice and provided evidence that both TNF receptors (1 and 2) are expressed in mouse oocytes, with TNF receptor 2 as an important mediator of TNF-α activity in the ovary^19^. Notably, mice lacking TNF-α exhibit prolonged fertility, accompanied with elevated primordial follicle numbers and enhanced ovulation rate and litter size^48^. However, whether these phenotypes are related to an increased follicular endowment at birth, as opposed to reduced follicle loss during ageing is not certain. Interestingly, a genome wide association study to identify loci associated with age at menopause, suggested that TNF-α, and susceptibility to inflammation, could impact on ovarian ageing^49^. Overall, these data suggest that there may be a relationship between inflammatory levels, apoptosis and ovarian ageing. Some studies have also indicted that cytokines may also modulate oocyte quality. For example, while deletion of IL-1α in mice did not increase follicle numbers in 12-month-old mice compared to controls, it did improve ovulation rate, pregnancy and litter size, highlighting the potential impact of cytokines on fertility^41^. However, which specific molecular processes involved are yet to be described in any detail.

In addition to this age-associated increase in the pro-inflammatory status, an increase in some innate and adaptive immune cells was observed in the ageing ovary. Our results revealed an increased percentage of macrophages at 12 months of age compared to 2-month-old mice. This is particularly relevant as macrophages are the most prominent immune cells within the mammalian ovary and are critical for the correct ovarian function and optimal fertility because of the cytokines, chemokines and growth factors they secrete^20,50^. Macrophages detected in the ovary can be either considered tissue-resident cells or monocyte-derived macrophage that infiltrate the ovary. A recent study in mice demonstrated that the proportion of monocyte-derived macrophages increased dramatically in the aged ovaries, while tissue-resident macrophages decreased^15^. This phenomenon was accompanied by a significant increase in the expression levels of ccl5, a chemokine involved in leukocyte recruitment to inflammation sites^15^. We also observed increased ccl5 expression, suggesting increased proportion of macrophages in the ovary could be in consequence to enhanced infiltration of circulating monocytes.

In contrast, in the same study, a decrease in the proportion of total ovarian macrophages was observed with age^15^. This is unexpected as we observed the opposite scenario. These discrepancies could be attributed to differences in the sample processing for flow cytometry, or they may be related to differences in the estrous cycle, during which macrophage number and distribution can change^20^. Briley et al.^14^ quantified macrophages in ovarian sections from CB6F1 mice (different to the strain used in our study) using an antibody against F4/80, and found similar numbers of F4/80-positive cells in ovaries of reproductively young and old mice (23.9% ± 1.7% and 25.4% ± 0.83% of total cells, respectively). However, they also reported the presence of macrophage giant cells in ovaries at advanced reproductive age that were absent in ovaries from young mice, similar to Asano et al.^51^, suggesting changes in the macrophage morphology are associated with ovarian ageing^14^.

Interestingly, the percentage of total B cells, as defined by CD19 expression, was also significantly increased in ovaries from 6 and 18-month-old mice, compared to 2-month-old mice. While the total number of B cells decreases with age in humans, age related changes in the percentage of specific B cells subsets are less well characterised^52^. It is possible that the B cells we detected in the ovary are tissue-resident memory B (BRM) cells, which are maintained without recirculating in non-lymphoid tissues^53^. Unfortunately, a specific marker for BRM cells in mice is not yet available, so we are unable to test this hypothesis. BRM cells have been found in the lungs^54^, skin^55^ and intestinal tract^56^, where their role is to effectively and quickly produce antibodies following secondary antigenic stimulation^53^. However, accumulation of memory B cells in peripheral tissues, along with a decreased B cell production in the bone marrow with age, leads to a reduced diversity and limited response to new antigens^57^. B cells can also release inflammatory cytokines like CSF2^58^, or anti-inflammatory cytokines, such as IL-10^59^. Therefore, infiltration of memory B cells within the ovary during ageing could contribute to the pro-inflammatory environment.

Furthermore, our results indicate infiltration of CD4 + cells with age in the ovary, while percentage of CD8 + cell population did not change. Consistent with these findings, Saule et al. also observed an increase in T memory cells in aged people^60^. Interaction with B cells have shown to be necessary for the maintenance of a long-lived population of CD4 + memory cells in the lungs^61,62^. Communication between CD4 + lymphocytes and B cells could also occur in the ovary, as both populations were significantly increased at 18 months of age. In the female reproductive tract, Iijima et al. showed that CD4 + memory cells form clusters with other resident immune cells, such as CD8 + T cells and macrophages, to enable an effective immune response and ensure cell maintenance after intravaginal infection^63^. Infiltration of CD4 + cells could be involved in the maintenance of an inflammatory microenvironment in the aged ovary by secreting different cytokines, TNF-α being one of the most commonly studied. However, more studies are required to confirm if a relationship such as this is functionally relevant in the ovary.

Another subset of T cells, termed regulatory T cells (Treg), are also recruited to sites of inflammation in peripheral tissues in order to resolve inflammation and restore the correct organ function^64^. Recently, Dong et al. suggested some natural regulation of ovarian integrity by Tregs, since depletion of Treg cells by neonatal thymectomy results in autoimmune ovarian disease^65^. Therefore, the infiltrating CD4 + T cells observed with ageing could be Tregs that have moved to the ovary to counteract inflammation. This could be further investigated by staining CD4 + cells for FOXP3, which is the definitive marker that identifies Tregs.

The data presented in this study provides novel characterization of inflammation in normal follicle depletion that occurs as females age. Our findings indicate ovarian ageing is associated with increased percentage in intra-ovarian CD4 + T cells, B cells and macrophages. Levels of several cytokines and chemokines are increased in the aged ovary. Macrophages could be playing a critical role in the ovary due to their major role in cytokine secretion, as some of these cytokines and TNF-α in particular, are known to induce ovarian follicle death. Overall, the results observed in this study provide a step towards improving understanding of the potential mechanisms responsible for the regulation of oocyte number as female age. However, much work is necessary to determine how alterations in immune cells and their modulation of the ovarian inflammatory environment might mediate follicle loss over the course of reproductive lifespan.

## Supplementary Information

Supplementary information.

## Data Availability

Materials, data and associated protocols are available on request.

## References

[1] Broekmans FJ, Soules MR, Fauser BC (2009). Ovarian aging: mechanisms and clinical consequences. Endocr. Rev..

[2] Findlay JK, Hutt KJ, Hickey M, Anderson RA (2018). How is the number of primordial follicles in the ovarian reserve established?. Biol. Reprod..

[3] McGee EA (2004). Initial and cyclic recruitment of ovarian follicles. Endocr. Rev..

[4] Findlay JK, Hutt KJ, Hickey M, Anderson RA (2015). What is the ‘ovarian reserve’?. Fertil. Steril..

[5] Balasch J, Gratacos E (2012). Delayed childbearing: effects on fertility and the outcome of pregnancy. Curr. Opin. Obs. Gynecol..

[6] Franceschi C, Campisi J (2014). Chronic inflammation (inflammaging) and its potential contribution to age-associated diseases. J. Gerontol. Ser. A Biol. Sci. Med. Sci..

[7] Xia, S. *et al.* An Update on inflamm-aging: mechanisms, prevention, and treatment. *J. Immunol. Res.* **2016** (2016). 10.1155/2016/8426874 PMC4963991 27493973

[8] Goldberg EL, Dixit VD (2015). Drivers of age-related inflammation and strategies for healthspan extension. Immunol. Rev..

[9] Maggio M, Guralnik JM, Longo DL, Ferrucci L (2006). Interleukin-6 in aging and chronic disease: a magnificent pathway. J. Gerontol. Med. Sci..

[10] Pedersen M (2003). Circulating levels of TNF-alpha and IL-6-relation to truncal fat mass and muscle mass in healthy elderly individuals and in patients with type-2 diabetes. Mech. Ageing Dev..

[11] Bruunsgaard H, Andersen-Ranberg K, Hjelmborg JVB, Pedersen BK, Jeune B (2003). Elevated levels of tumor necrosis factor alpha and mortality in centenarians. Am. J. Med..

[12] Cesari M (2004). Inflammatory markers and physical performance in older persons: the InCHIANTI study. J. Gerontol. Ser. A.

[13] Youm YH (2013). Canonical Nlrp3 inflammasome links systemic low-grade inflammation to functional decline in aging. Cell Metab..

[14] Briley SM (2016). Reproductive age-associated fibrosis in the stroma of the mammalian ovary. Reproduction.

[15] Zhang Z, Schlamp F, Huang L, Clark H, Brayboy L (2020). Inflammaging is associated with shifted macrophage ontogeny and polarization in the aging mouse ovary. Reproduction.

[16] Jabbour HN, Sales KJ, Catalano RD, Norman JE (2009). Inflammatory pathways in female reproductive health and disease. Reproduction.

[17] Roby KF, Weed J, Lyles R, Terranova PF (1990). Immunological evidence for a human ovarian tumor necrosis factor-α. J. Clin. Endocrinol. Metab..

[18] Chen HL, Marcinkiewicz JL, Sancho-Tello M, Hunt JS, Terranova PF (1993). Tumor necrosis factor-alpha gene expression in mouse oocytes and follicular cells. Biol. Reprod..

[19] Greenfeld CR (2007). Tumor necrosis factor (TNF) receptor type 2 is an important mediator of TNF alpha function in the mouse ovary. Biol. Reprod..

[20] Wu R, Van der Hoek KH, Ryan NK, Norman RJ, Robker RL (2004). Macrophage contributions to ovarian function. Hum. Reprod. Update.

[21] Weiss G, Goldsmith LT, Taylor RN, Bellet D, Taylor HS (2009). Inflammation in reproductive disorders. Reprod. Sci..

[22] Herath S (2007). Ovarian follicular cells have innate immune capabilities that modulate their endocrine function. Reproduction.

[23] Henes M (2015). Ovarian reserve alterations in premenopausal women with chronic inflammatory rheumatic diseases: impact of rheumatoid arthritis, Behcet’s disease and spondyloarthritis on anti-Mullerian hormone levels. Rheumatology.

[24] Fréour T (2012). Ovarian reserve in young women of reproductive age with Crohn’s disease. Inflamm. Bowel Dis..

[25] Şenateş E (2013). Serum anti-Müllerian hormone levels are lower in reproductive-age women with Crohn’s disease compared to healthy control women. J. Crohn’s Colitis.

[26] de Souza FHC (2015). Reduced ovarian reserve in patients with adult polymyositis. Clin. Rheumatol..

[27] Snider AP, Wood JR (2019). Obesity induces ovarian inflammation and reduces oocyte quality. Reproduction.

[28] Ora MICC, Ooistra LIK, Travlos G (2015). Vaginal cytology of the laboratory rat and mouse: review and criteria for the staging of the Estrous cycle using stained vaginal smears. Toxicol. Pathol..

[29] Myers M, Britt KL, Wreford NGM, Ebling FJP, Kerr JB (2004). Methods for quantifying follicular numbers within the mouse ovary. Reproduction.

[30] Liew SH (2014). Loss of the proapoptotic bh3-only protein bcl-2 modifying factor prolongs the fertile life span in female mice1. Biol. Reprod..

[31] Geuna S, Herrera-Rincon C (2015). Update on stereology for light microscopy. Cell Tissue Res..

[32] Liew SH, Nguyen QN, Strasser A, Findlay JK, Hutt KJ (2017). The ovarian reserve is depleted during puberty in a hormonally driven process dependent on the pro-Apoptotic protein BMF. Cell Death Dis..

[33] Berkholtz CB, Lai BE, Woodruff TK, Shea LD (2006). Distribution of extracellular matrix proteins type I collagen, type IV collagen, fibronectin and laminin in mouse folliculogenesis. Histochem. Cell Biol..

[34] Rea IM (2018). Age and age-related diseases: role of inflammation triggers and cytokines. Front. Immunol..

[35] Comi M, Amodio G, Gregori S (2018). Interleukin-10-producing DC-10 Is a unique tool to promote tolerance via antigen-specific t regulatory type 1 cells. Front. Immunol..

[36] Gabay C (2006). Interleukin-6 and chronic inflammation. Arthritis Res. Ther..

[37] Song F, Ma Y, Bai X, Chen X (2016). The expression changes of inflammasomes in the aging rat kidneys. J. Gerontol. Biol. Sci..

[38] Reilly, S. O., Cant, R., Ciechomska, M. & Laar, J. M. Van. Interleukin-6: a new therapeutic target in systemic sclerosis? *Clin. Transl. Immunol.* **2** (2013). 10.1038/cti.2013.2 PMC4232056 25505952

[39] Zhou F, Shi L, Zhang S (2017). Ovarian fibrosis: a phenomenon of concern. Chin. Med. J. (Engl).

[40] Wood GA, Fata JE, Watson KLM, Khokha R (2007). Circulating hormones and estrous stage predict cellular and stromal remodeling in murine uterus. Reproduction.

[41] Uri-Belapolsky S (2014). Interleukin-1 deficiency prolongs ovarian lifespan in mice. Proc. Natl. Acad. Sci..

[42] Latz E, Duewell P (2018). NLRP3 inflammasome activation in inflammaging. Semin. Immunol..

[43] Tsuji-takayama K, Aizawa Y, Okamoto I, Kojima H, Koide K (1999). Interleukin-18 Induces Interferon-gamma production through NF-kB and NFAT Activation in Murine T Helper Type 1 Cells. Cell. Immunol..

[44] Liu, T., Zhang, L., Joo, D. & Sun, S. NF-κB signaling in inflammation. *Signal Transduct. Target. Ther.* **2** (2017). 10.1038/sigtrans.2017.23 PMC5661633 29158945

[45] Terranova PF (1997). Potential roles of tumor necrosis factor-α in follicular development, ovulation, and the life span of the corpus luteum. Domest. Anim. Endocrinol..

[46] Kaipia A, Chun S, Eisenhauer K, Hsueh AJW (1996). Tumor necrosis factor-a and its second messenger, ceramide, stimulate apoptosis in cultures ovarian follicles. Endocrinology.

[47] Morrison LJ, Marcinkiewicz JL (2002). Tumor necrosis factor alpha enhances oocyte/follicle apoptosis in the neonatal rat ovary. Biol. Reprod..

[48] Cui L, Yang G, Pan J, Zhang C (2011). Tumor necrosis factor alpha knockout increases fertility of mice. Theriogenology.

[49] Stolk L (2012). Meta-analyses identify 13 loci associated with age at menopause and highlight DNA repair and immune pathways. Nat. Genet..

[50] Tingen CM (2014). A macrophage and theca cell-enriched stromal cell population influences growth and survival of immature murine follicles in vitro. Reproduction.

[51] Asano Y (2012). Age-related accumulation of non-heme ferric and ferrous iron in mouse ovarian stroma visualized by sensitive non-heme iron histochemistry. J. Histochem. Cytochem..

[52] Wu AAY, Dunn-walters D (2010). The ageing B cell population: composition and function. Biogerontology.

[53] Allie SR, Randall TD (2020). Resident memory B cells. Viral Immunol..

[54] Allie, S. R. *et al.* The establishment of resident memory B cells in the lung requires local antigen encounter. *Nat. Immunol.* **20** (2019). 10.1038/s41590-018-0260-6 PMC6392030 30510223

[55] Egbuniwe IU, Karagiannis SN, Nestle FO, Lacy KE (2015). Revisiting the role of B cells in skin immune surveillance. Trends Immunol..

[56] Reboldi A, Cyster JG, Francisco CS, Francisco S (2017). Peyer’s patches: Organizing B cell responses at the intestinal frontier. Immunol Rev.

[57] Linehan, E. & Fitzgerald, D. C. Ageing and the immune system: focus on macrophages. *Eur. J. Microbiol. Immunol.* **5** (2015). 10.1556/EUJMI-D-14-00035 PMC4397845 25883791

[58] Weber GF (2014). Pleural innate response activator B cells protect against pneumonia via a GM-CSF-IgM axis. J. Exp. Med..

[59] Madan R (2009). Nonredundant roles for B cell-derived IL-10 in immune counter-regulation. J Immunol.

[60] Saule P, Trauet J, Dutriez V, Dessaint J, Labalette M (2006). Accumulation of memory T cells from childhood to old age: Central and effector memory cells in CD4 + versus effector memory and terminally differentiated memory cells in CD8 + compartment. Mech. Ageing Dev..

[61] Hondowicz BD, Kim KS, Ruterbusch MJ, Keitany GJ (2018). IL-2 is required for the generation of viral-specific CD4+ Th1 Trms cells and B cells are essential for maintenance in the lung. Eur. J. Immunol..

[62] Strutt TM (2017). IL-15 supports the generation of protective lung-resident memory CD4 T cells. Nat. Publ. Gr..

[63] Iijima N (2008). Dendritic cells and B cells maximize mucosal Th1 memory response to herpes simplex virus. J. Exp. Med..

[64] Korn T, Muschaweckh A (2019). Stability and maintenance of Foxp3 + treg cells in non-lymphoid microenvironments. Front. Immunol..

[65] Dong Y (2016). The role of regulatory T cells in thymectomy-induced autoimmune ovarian disease. Am. J. Reprod. Immunol..

