# Supplementary material for: Evaluation of inflammation and follicle depletion during ovarian ageing in mice
Source: Sci Rep. 2021 Jan 11;11:278. doi: 10.1038/s41598-020-79488-4 (PMC7801638; doi:10.1038/s41598-020-79488-4)
Supplement: Supplementary file 1 — Supplementary information. [file 41598_2020_79488_MOESM1_ESM.pdf]

Title: Evaluation of inflammation and follicle depletion during ovarian ageing in mice

Authors: Carolina Lliberos[1], Seng H Liew[1], Pirooz Zareie[2], Nicole L La Gruta[2], Ashley Mansell[3], Karla Hutt[1]*

Corresponding author: Karla Hutt
[1]Department of Anatomy and Developmental Biology, Monash Biomedicine Discovery Institute, Monash University, Clayton, Victoria, Australia.
[2]Department of Biochemistry and Molecular Biology, Monash Biomedicine Discovery Institute, Monash University, Clayton, Victoria, Australia.
[3]Centre for Innate Immunity and Infectious Diseases, Hudson Institute of Medical Research, Clayton, Victoria, Australia.
*Corresponding author e-mail: karla.hutt@monash.edu

Supplemental Figure 1

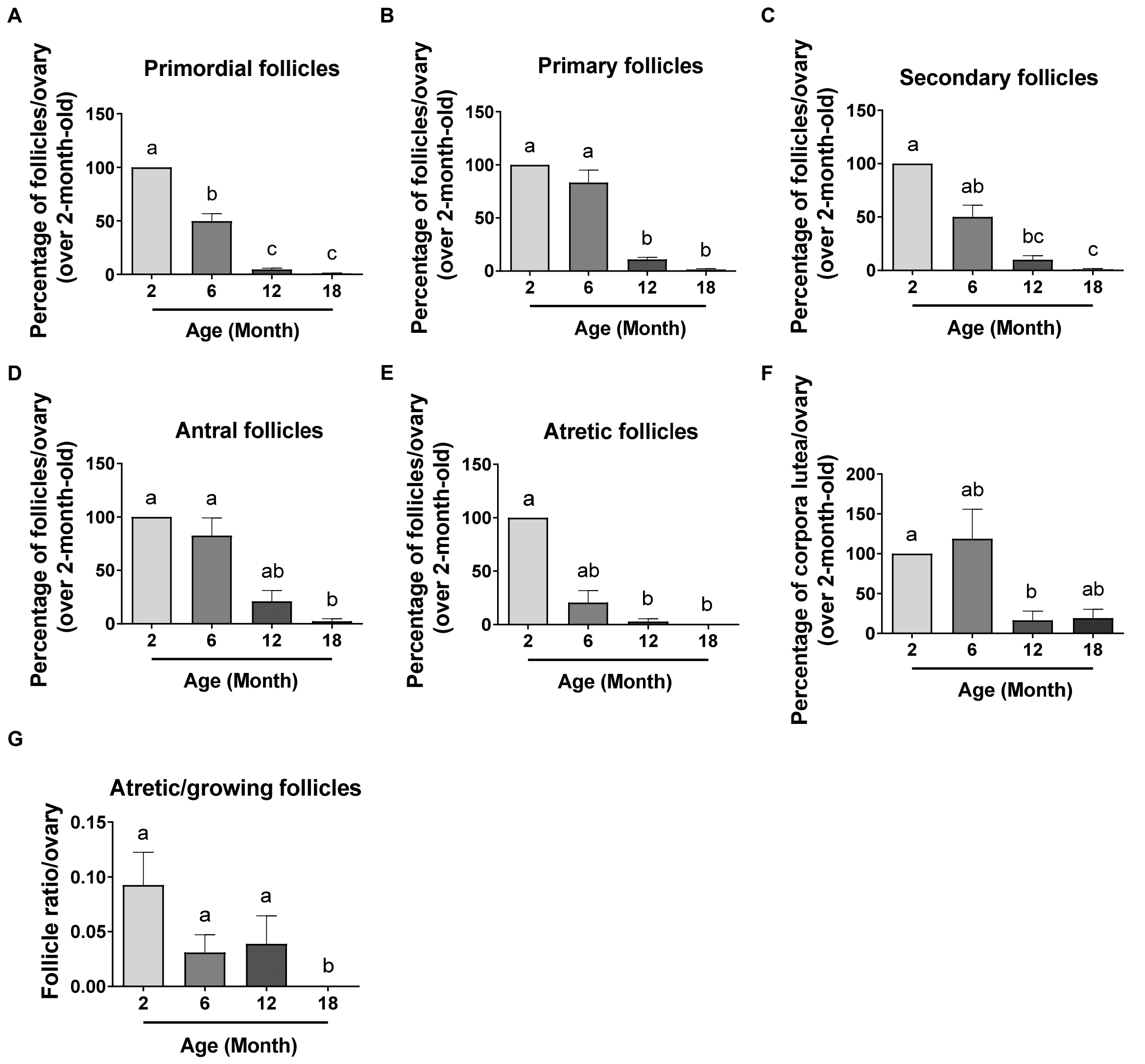

Supplemental Figure 1. Primordial (A), primary (B), secondary (C), antral (D), atretic follicle (E) and corpora lutea (F) percentage (relative to 2-month-old group) and ratio of atretic follicles per total number of growing follicles (secondary and antral follicles) (G) in ovaries from 2, 6, 12 and 18-month-old mice. n = 6 per cohort. Data are presented as mean±SEM; Kruskal-Wallis test, Dunn’s multiple comparisons test: a, b and c are significantly different among groups ($p<0.05$); Ordinary One-way ANOVA, Tukey’s multiple comparisons test: a, b and c are significantly different among groups ($p<0.05$).

Supplemental Figure 2

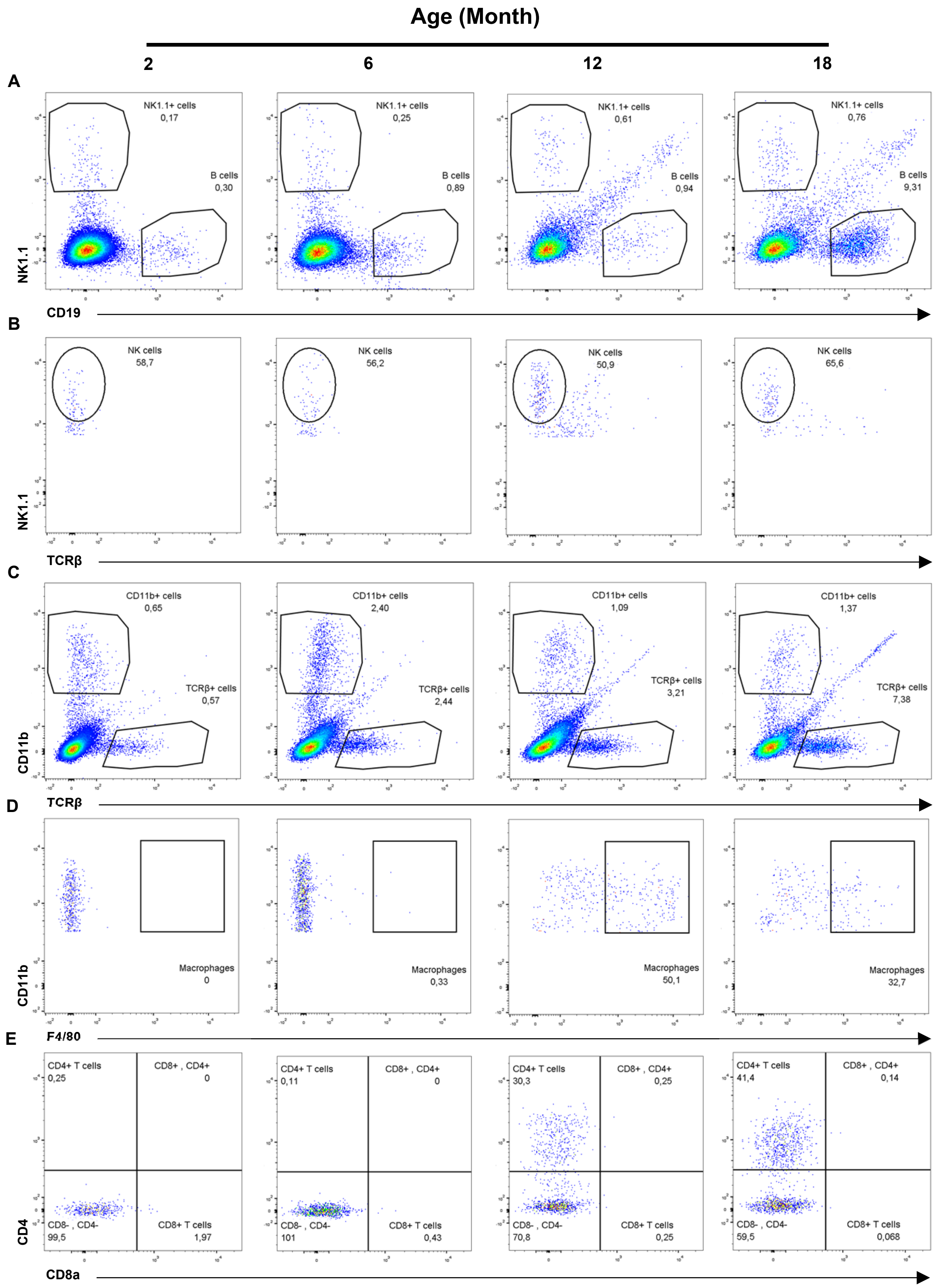

Supplemental Figure 2. Representative dot plots of B cells (A), NK1.1+TCRβ- NK cells (B), CD11b+ and T cells (C), F4/80+CD11b+ macrophages (D) and CD4+ and CD8+ T cells (E) identified in ovaries from 2, 6, 12 and 18-month-old mice by Flow cytometry. n = 3-5 per cohort.

Supplemental Figure 3

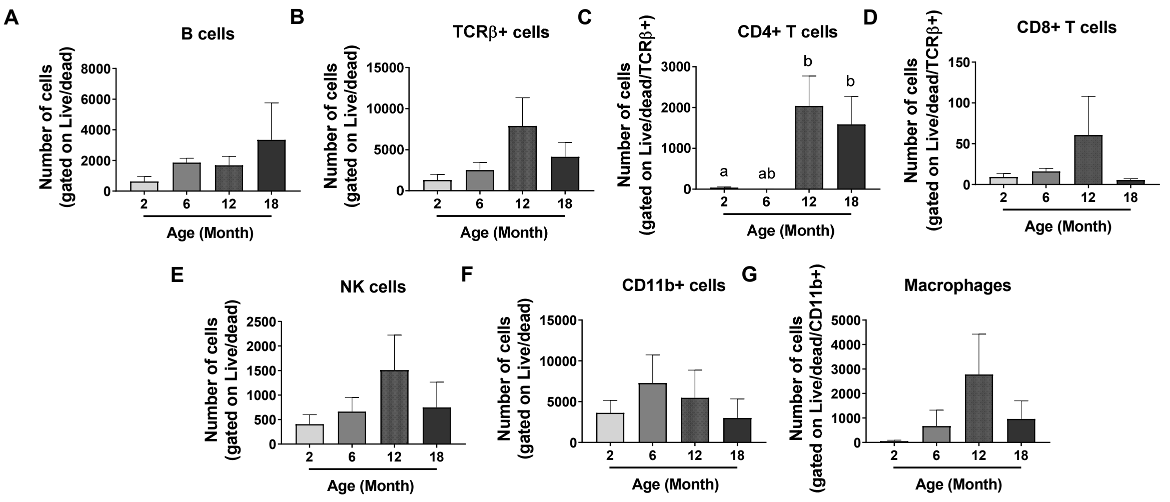

Supplemental Figure 3. Number of B cells (A), T cells (B), CD4+ (C) and CD8+ T cells (D), NK1.1+TCRβ- NK cells (E), CD11b+ cells (F) and F4/80+CD11b+ macrophages (G) identified in ovaries from 2, 6, 12 and 18-month-old mice. n = 3-5 per cohort. Data are presented as mean±SEM; A, B, E, F and G: Kruskal-Wallis test, Dunn's multiple comparisons test ($p>0.05$); C and D: Ordinary One-way ANOVA, Tukey's multiple comparisons test: a and b are significantly different among groups ($p<0.05$).

Supplemental Figure 4

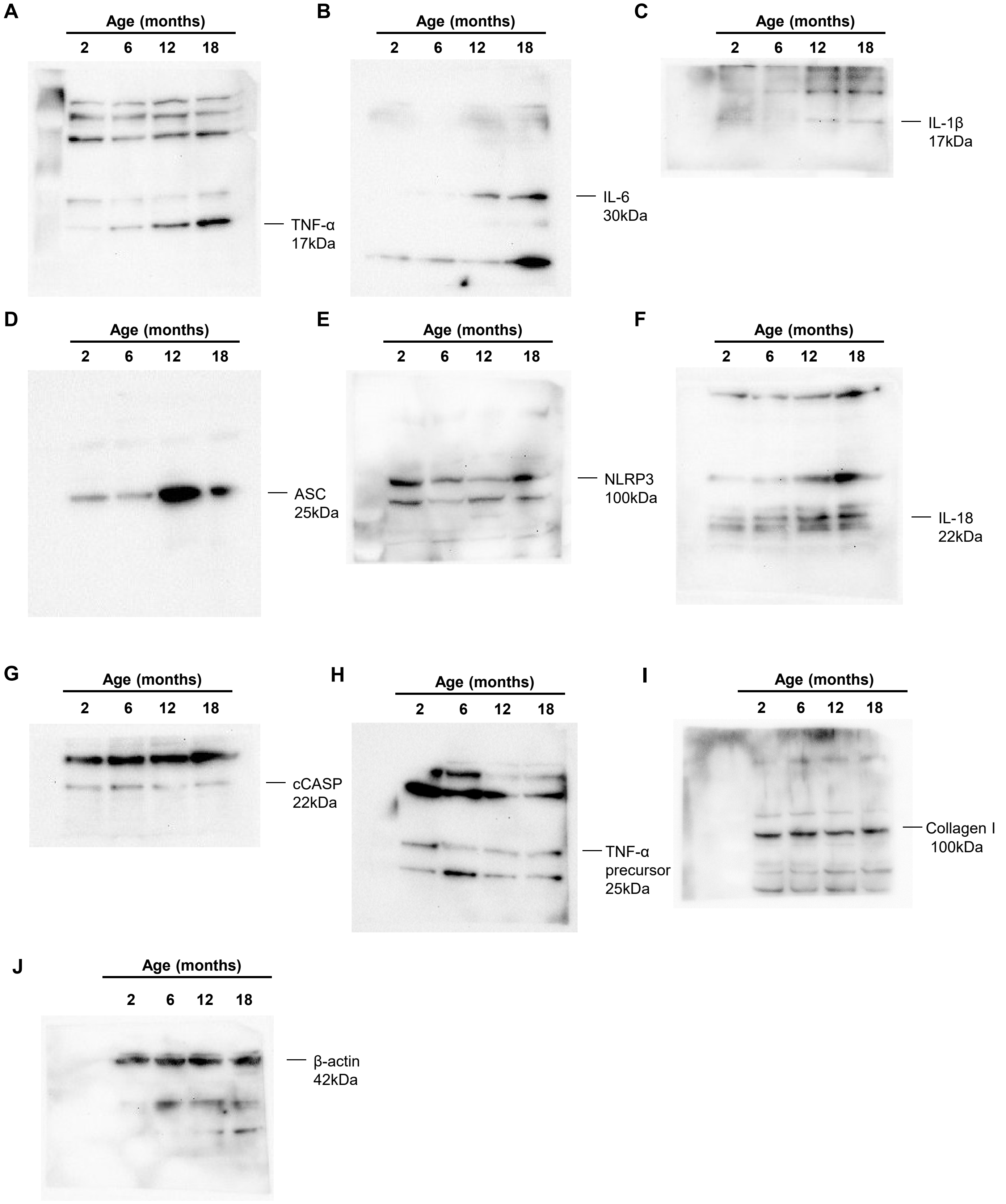

Supplemental Figure 4. Representative images of full length Western blots showing ovarian protein levels of TNF-α (A), IL-6 (B), IL-1β (C), ASC (D), NLRP3 (E), IL-18 (F), cleaved Casp1 (G), TNF-α precursor (H), collagen I (I) and β-actin (J) from 2, 6, 12 and 18-month-old mice.

Supplemental Table 1. Stage of estrous cycle

Mice age (Months)	Stage of estrous cycle
2	Estrus
	Estrus
	Estrus
	Estrus
	Diestrus
6	Diestrus
	Diestrus/Proestrus
	Diestrus
	Diestrus
12	Diestrus
	Diestrus
	Diestrus
18	Metestrus
	Diestrus
	Estrus

Supplemental Table 2. List of primers used for quantitative real-time PCR

Gene	Sequence (5'→3')	PCR product (bp)
Asc (PYD and CARD domain containing)	F: GACAGTACCAGGCAGTTCGT<br>R: AGTCCTTGCAGGTCAGGTTC	96
Gapdh (glyceraldehyde-3-phosphate dehydrogenase)	F: TCCATGACAACTTTGGCATTG<br>R: CAGTCTTCTGGGTGGCAGTGA	72
Casp1 (Caspase 1)	F: CACGCCCTGTTGGAAAGGAA<br>R: CCCTCAGGATCTTGTCAGCCA	94
Ccl5 (Chemokine (C-C motif) ligand 5)	F: TTTGCCTACCTCTCCCTCG<br>R: CGACTGCAAGATTGGAGCACT	85
Col1a1 (Collagen, type I, alpha 1)	F: CCGATGGATTCCCGTTCGAG<br>R: GAGGCCTCGGTGGACATTAG	97
Col4a1 (Collagen, type IV, alpha 1)	F: GCGTAAGTTCAGCACCATGC<br>R: CACAAACCGCACACCTGCTA	173
Csf1 (Colony stimulating factor 1)	F: GAACAAGGCCTGTGTCCGAA<br>R: CCACATCTCGGCTAGAGCAC	161
Csf2 (Colony-stimulating factor 2)	F: AACTCCGGAAACGGACTGTG<br>R: AAGCTGGATTCAGAGCTGGC	145
Il10 (Interleukin 10)	F: GCTCTTACTGACTGGCATGAG<br>R: CGCAGCTCTAGGAGCATGTG	105
Il18 (Interleukin 18)	F: CCTTTGAGGCATCCAGGACA<br>R: GGGAACAGCCAGTGTTCAGT	87
Il1a (Interleukin 1 alpha)	F: GTATGCCTACTCGTCGGGAG<br>R: GGCAACTCCTTCAGCAACAC	120
Il1b (Interleukin 1 beta)	F: GAAGAAGAGCCCATCCTCTG<br>R: GGAGCCTGTAGTGCAGTTGT	91
Il6 (Interleukin 6)	F: CTGCAAGAGACTTCCATCCAG<br>R: AGTGGTATAGACAGGTCTGTTGG	131
Nlrp3 (NLR family, pyrin domain containing 3)	F: CAGAGCCTACAGTTGGGTGA<br>R: TAGCAGTGAAGAGCAGTGCG	193
Tnfa (Tumor necrosis factor alpha)	F: CCTGGCCTCTCTACCTTGTTG<br>R: AGCCTGGTCACCAAATCAGC	100

Supplemental Table 3. Panel of antibodies and other stains used for Flow Cytometry

Antibody	Conjugate	Clone	Specificity	Concentration	Company
CD19	BV650	1D3	B cells	1:400	BD Biosciences, 563235
CD4	BUV395	GK1.5	$CD4^+$ T cells	1:400	BD Biosciences, 563790
CD8a	PerCP-Cy5.5	53-6.7	$CD8^+$ T cells	1:400	BD Biosciences, 551162
CD11b	FITC	M1/70	$CD11b^+$ cells	1:400	BD Biosciences, 557396
F4/80	PE	T45-2342	Macrophages	1:200	BD Biosciences, 565410
NK1.1	PE-Cy7	PK136	NK cells	1:600	BD Biosciences, 552878
TCRβ	BV510	H57-597	T cells	1:400	BD Biosciences, 563221
Live/Dead Viability Stain 700	FVS700	-	Dead cells	1:2000	BD Biosciences, 564997
